# Changes in Medical Interns’ Psychological Well-Being and Work Investment Perceptions During the Internship Year: An Interim Prospective Repeated-Measures Study at a Single Tertiary Medical Center

**DOI:** 10.3390/healthcare14193175

**Published:** 2026-09-24

**Authors:** Marwa Sabih, Guy Vishnevsky, Yara Hamoud, Hanni Robinson, Shachar Keinan, Noam Klegar, Gad Segal, Vered Robinzon, Uri Manor, Raphael Snir, Reut Shoham

**Affiliations:** 1 Education Authority, The Chaim Sheba Medical Center, Tel-Hashomer, Ramat Gan 52621, Israel; 2 Department of Internal Medicine H, Tel Aviv Sourasky Medical Center, Tel Aviv 64239, Israel; 3 Gray Faculty of Medical & Health Sciences, Tel Aviv University, Tel Aviv 69978, Israel; 4 Department of Clinical Dermatology, Hadassa Medical Center, Jerusalem 91120, Israel; 5 School of Medicine, University of Nicosia, Nicosia 2408, Cyprus; 6 Faculty of Health Science Management, Ariel University, Ariel 44837, Israel; 7 Management Wing, The Chaim Sheba Medical Center, Tel-Hashomer, Ramat Gan 52621, Israel; 8 The Academic College of Tel Aviv-Yafo, Tel Aviv 61083, Israel; rafi-snir@bezeqint.net

**Keywords:** satisfaction, work investment, interns, motivation, organizational culture

## Abstract

**Background:** Medical internship is a demanding transition into supervised clinical responsibility, but longitudinal evidence on interns’ well-being and work investment remains limited. **Objective:** To examine change across Q1–Q3 in self-reported well-being, work investment, and organizational perceptions. **Methods:** This prospective interim study included 126 questionnaires from 42 interns. The prespecified primary endpoint was change in general life satisfaction, assessed using the five-item Satisfaction With Life Scale (SWLS). Secondary repeated-measures outcomes were work satisfaction, work enjoyment, perceived stress, emotional and physical fatigue, weekly work hours, and perceived organizational culture. Repeated-measures ANOVA assessed omnibus time effects; linear contrasts were reported separately. Exploratory analyses used one mean score per intern. **Results:** Among 41 interns with complete SWLS scores across quarters, means were 4.36 ± 1.24, 4.56 ± 1.18, and 4.72 ± 1.04 on the 1–7 scale. The omnibus time effect did not reach statistical significance (F[2,80] = 3.005, *p* = 0.055, partial η^2^ = 0.070), although the linear contrast was nominally significant (*p* = 0.021); the Q3–Q1 difference was 0.36 points (95% CI 0.06–0.67). Weekly work hours increased from 58.98 ± 12.25 to 62.98 ± 11.82. Their omnibus effect was not significant (F[2,82] = 2.726, *p* = 0.071, partial η^2^ = 0.062), whereas the linear contrast was nominally significant (F[1,41] = 4.954, *p* = 0.032, partial η^2^ = 0.108). Other prespecified secondary omnibus tests were not significant. Internal consistency varied across measures and quarters. Exploratory participant-level comparisons indicated lower stress and physical fatigue among males and several inter-domain associations. **Conclusions:** No significant omnibus change was detected in the primary endpoint or the prespecified secondary outcomes. Nominal upward linear trends in life satisfaction and work hours warrant further investigation. Our findings should be regarded as preliminary and hypothesis-generating, rather than as evidence of sustained stability, resilience, or causal adaptation throughout internship.

## 1. Background

In Israel, the internship year, referred to as the *stage* year, is the final stage of academic and clinical training before medical graduates receive their license to practice. Comparable to Foundation Year 1 in the United Kingdom [1] and postgraduate year 1 in the United States [2], this 12-month period is widely recognized as physically, cognitively, and emotionally demanding [3,4]. After six or seven years of medical school, Israeli interns rotate through major clinical departments and, for the first time, assume direct responsibility for patients under supervision. These rotations involve frequent changes in clinical environment, prolonged shifts, sleep restriction, emotionally difficult encounters, and decisions about future specialty training. Together, these factors create a plausible context for changes in well-being, work investment, motivation, and perceptions of organizational culture during internship.

Due to the intense workload of hospital internships, interns can be classified as individuals who invest heavily in their work. Snir and Harpaz [5,6] identified four subtypes of such employees: workaholics, work enthusiasts, organization-directed, and those motivated by pressing financial needs. A recent study by Snir and colleagues [7] at the Executive Survey Institute, Sheba Medical Center, found that individuals belonging to these groups are at increased risk for health issues, likely stemming from sustained mental and physiological stress. Notably, work enthusiasts were found to be at even greater risk of heart disease than both workaholics and regular full-time workers.

Schaufeli and co. [8] illuminated the association between workaholism (defined as working excessively and compulsively), burnout and well-being among junior physicians, a group known to be susceptible to workaholism. Among these young physicians, workaholism led to increased burnout and poor well-being. A global meta-analysis [9] emphasized the prevalence and importance of burnout in this group, reporting a burnout prevalence of 47.3% among postgraduate medical trainees. More recently, Chaillet and co. [10] demonstrated that both high perceived stress and long weekly work hours significantly increase the risk of workaholism among interns. Moreover, it was found [11] that over a quarter of newly qualified doctors screened positive for pathological anxiety at the start of their internship year. This anxiety was particularly pronounced among female doctors and those aged 21 to 25 years.

The psychological burden faced by junior physicians is amplified by unique occupational stressors. Key contributors to intern stress were identified by Antoniou and co. [12], including the potential for serious medical errors, excessive work hours, and inadequate psychological support. They also highlighted the impact of role ambiguity; job demands and limited financial resources on increasing stress and reducing job satisfaction. Importantly, their study found that female interns reported significantly higher stress and lower job satisfaction compared to their male counterparts, suggesting that gender plays a critical role in the intern experience.

Given these circumstances, it is only plausible to assume that the demands of internship may lead to changes in an individual’s pattern of work investment. For example, repeated exposure to high-stress departments that offer minimal personal support might cause a shift from work enthusiasm to organization-directed behavior. These changes in work investment may also be reflected in fluctuations in psychological variables such as perceived stress and overall well-being. Moreover, it is important to consider whether such changes differ by gender, as research has consistently shown that female doctors experience higher levels of stress and burnout compared to their male counterparts [12,13]. The gender differences in workaholism, stress management and different coping mechanisms are well described [14,15].

### Aim of the Current Study

The current interim analysis was designed to describe selected psychological, behavioral, and organizational variables among medical interns across the early internship year. The primary repeated-measures question was whether general life satisfaction changed between three consecutive quarterly assessments. Secondary repeated-measures questions examined changes in work satisfaction, work enjoyment, perceived stress, fatigue, weekly work hours, and perceived organizational culture. Exploratory analyses examined associations with age, sex, relationship status, work hours, fatigue, satisfaction, affective well-being, work investment, and perceived organizational culture. We anticipated that the demands of internship might be associated with deterioration in some well-being indicators; however, given the interim sample size and the exploratory scope of the study, all secondary and exploratory associations were treated as hypothesis-generating.

## 2. Materials and Methods

### 2.1. Participants and Study Design

Interns beginning their internship year at Sheba Medical Center, Israel, during 2024–2025, were invited to participate. The study was approved by the local Institutional Review Board at the Chaim Sheba Medical Center (approval # SMC-23-0565) and was conducted in accordance with the Declaration of Helsinki. After providing written informed consent, participants completed a structured questionnaire at the beginning of internship and then at three-month intervals. The parent study was designed as a prospective repeated-measures study with up to four planned measurements per participant across the internship year.

The present manuscript reports an interim analysis restricted to participants who had completed three consecutive quarterly questionnaires by 30 April 2025. Participants who did not complete all three included questionnaires or who withdrew consent were excluded from this interim dataset. Accordingly, the analytic sample comprised repeated observations nested within 42 interns rather than independent cross-sectional observations.

### 2.2. Research Instrument

The questionnaire collected demographic and contextual information, including age, sex, religion, country of birth, parental country of origin, number of dependents, relationship status, partner work hours when applicable, average weekly work hours, average sleep duration, and whether participants took extra on-call shifts because of financial strain. Participants were also asked whether they would stop working if they hypothetically won the lottery or inherited a large sum of money. Emotional and physical fatigue were each represented by a ten-item binary symptom index, expressed as ten times the proportion of available symptoms endorsed; this equals a symptom count when all ten items are completed.

Additional questionnaire items were rated on 1–7 Likert scales and grouped conceptually into prespecified domains: effort and time investment, satisfaction and motivation, stress and affective well-being, fatigue, and organizational context. Work effort, time commitment, and work intensity were assessed using items adapted from Brown and Leigh [16]. Work enjoyment and work drive were assessed using the Spence and Robbins Workaholism Battery as validated in subsequent work [17]. Perceived high-demand/work-intensive organizational culture was assessed using items based on prior work on overwork and time demands [18]. General life satisfaction was assessed using the five Satisfaction With Life Scale items [19], reported as a mean on the 1–7 response scale rather than the conventional 5–35 sum, work-related affective well-being using Warr’s scale [20], work satisfaction using items based on Tsui and colleagues [21], perceived stress using Cohen’s perceived stress framework [22], and fatigue using symptom items informed by fatigue-related work [23]. These study implementations are not assumed to inherit the validation of the source instruments. Table 1 identifies the items, item counts, scoring, and internal consistency of every multi-item score analyzed. The supplementary questionnaire provides the complete administered wording.

Composite Likert scores were calculated as the arithmetic mean of available valid constituent items. Work enjoyment item Q7.6 was reverse-scored as 8 minus the response. The 14-item stress score used the same reversal for Q12.4, Q12.5, Q12.6, Q12.7, Q12.9, Q12.10, and Q12.13, followed by averaging all available items with equal weight. Positive and negative affect were scored separately using the six items specified in Table 1. The six-item significance/aptitude composite summarized perceived efficacy and contribution at work; it was not an objective competence assessment. Single-item measures, including work hours, sleep, leisure satisfaction, and work-intensity ratings, were analyzed separately and have no internal-consistency coefficient.

The emotional-fatigue index comprised decreased work efficiency, difficulty concentrating, decreased passion, nervousness, lack of perseverance, difficulty organizing thoughts, difficulty contacting people, lack of ambition, multiple mistakes at work, and decreased libido (Q13.1–Q13.10). The physical-fatigue index comprised eye fatigue, neck/shoulder stiffness, fatigue/drowsiness, headache, constipation/diarrhea, indigestion, physical pain, dizziness/ear ringing, rapid heartbeat/shortness of breath, and a tendency to catch colds (Q13.11–Q13.20). Each item was coded 0 or 1. Scores were calculated as 10 × the mean of available valid items, producing a prorated 0–10 symptom index when an item was missing. Higher scores indicated more symptoms. These were analyzed as study-specific symptom indices, not as established validated emotional- and physical-fatigue subscales or diagnostic measures. Internal consistency in this cohort is reported, but criterion validity, factor structure, and measurement invariance of these exact indices were not established in this population.

The item-level scoring audit identified a spreadsheet reference error in the stress composite: the Q12.8 reference pointed 51 rows below the questionnaire being scored. Stress was therefore recalculated directly from the participants’ own items. Eight entries outside their permitted item ranges were treated as missing rather than assigned guessed replacement values: two entries above 7 on 1–7 items, five zeros on 1–7 stress items, and one value of 4 on a binary fatigue item. All affected composites, descriptive summaries, and exploratory analyses were updated. The original raw entries were retained in the source file.

### 2.3. Statistical Analyses

Descriptive statistics are presented as mean ± standard deviation for continuous variables and as counts with percentages for categorical variables. Likert-scale items were summarized as continuous scores for descriptive and repeated-measures purposes. Pooled questionnaire-level summaries in Section 3.2 are descriptive only; the quarterly questionnaires were not treated as independent observations in the revised exploratory inference described below. The primary analytic focus was change across the three repeated quarterly assessments. Within-participant changes over time were analyzed using repeated-measures analysis of variance within a general linear model framework. Mauchly’s test was used to assess sphericity. When sphericity was violated, Greenhouse–Geisser correction was applied. For each repeated-measures outcome, analyses used interns with valid composite scores at all three quarters; the quarterly means and SDs in Table 2 describe that same outcome-specific sample. Partial η^2^ was calculated as the time-effect sum of squares divided by the sum of the time-effect and error sums of squares. Linear contrasts used coefficients −1, 0, and +1 across Q1–Q3; the corresponding F test has 1 and n − 1 degrees of freedom. Q3–Q1 mean differences and paired t-based 95% confidence intervals accompany these contrasts. The omnibus test evaluates any quarterly mean difference, whereas the linear contrast evaluates a specific ordered component. Contrasts and secondary endpoints were interpreted as nominal, unadjusted findings and did not replace the primary omnibus test.

For the purposes of interim analysis, the primary endpoint was prespecified as the within-participant change in general life satisfaction, measured by the Satisfaction With Life Scale, across Q1, Q2, and Q3. Secondary repeated-measures outcomes were work satisfaction, work enjoyment, perceived stress, emotional fatigue, physical fatigue, weekly work hours, and perceived high-demand organizational culture. Exploratory outcomes included demographic subgroup comparisons and inter-domain correlations. This hierarchy was used to reduce interpretive emphasis on isolated nominal findings from the broader exploratory analyses.

Internal consistency was estimated with raw-score Cronbach’s α separately at Q1, Q2, and Q3, using participants with complete constituent-item responses for the relevant scale at that quarter. Thus, each reliability estimate used independent interns rather than pooling repeated questionnaires. For binary fatigue items, this coefficient is equivalent to KR-20. Alpha values and their sample sizes are reported without deleting items to optimize reliability. Values below approximately 0.70 were interpreted as evidence of limited internal consistency, with attention to scale length and the small sample; alpha alone does not establish construct validity.

Secondary analyses examined associations between questionnaire domains and participant characteristics. To avoid pseudoreplication, all exploratory analyses in Section 3.3 were re-estimated using the participant as the independent unit of analysis. Each intern contributed one arithmetic mean of the available Q1–Q3 scores for each variable, requiring at least two valid quarterly scores; otherwise, that participant was excluded only from analyses involving that variable. Scores were reconstructed from the items using the scoring and range rules described above, and Excel error values were treated as missing. This aggregation estimates between-participant associations in average experience over the observation period; it does not estimate within-participant covariation or trajectories. Age, sex, and relationship status were taken from Q1 and verified against the original questionnaires, with missing baseline relationship status excluded from relationship-status comparisons. Between-group comparisons used Welch’s independent-samples *t* test on participant means, reporting mean differences and 95% confidence intervals; Mann–Whitney U tests were sensitivity analyses. Age associations and inter-domain associations used Spearman’s rank correlation on the participant-level dataset with pairwise exclusion of missing values; Pearson correlations were sensitivity checks. The analysis sample was 42 participants, except for organizational culture (41 participants), relationship-status comparisons (40 participants), and relationship status versus organizational culture (39 participants). Sensitivity analyses restricted group comparisons to participants with all three valid scores for the relevant outcome. Because the secondary analyses included numerous correlations and group comparisons in a small interim sample, these analyses were considered exploratory. No formal multiplicity correction was applied; therefore, isolated nominal *p*-values from secondary analyses should not be interpreted as confirmatory evidence. Emphasis was placed on the direction, magnitude, consistency, and plausibility of associations rather than on statistical significance alone.

The original analyses were performed using SPSS version 29 (IBM, Armonk, NY, USA). The revised scoring, reliability, longitudinal, and participant-level exploratory analyses were performed using Python 3.12.14 and SciPy 1.17.0. A two-sided *p* value lower than 0.05 was used to define nominal statistical significance, with the interpretive caveat noted above for the prespecified primary repeated-measures endpoint. Because multiple secondary and exploratory secondary analyses were conducted in a small interim sample, these results were not treated as confirmatory. They are presented to inform hypothesis development and planning of the full-cohort analysis rather than to establish definitive subgroup or correlational effects.

## 3. Results

### 3.1. Demographics

One hundred twenty-six questionnaires collected from 42 interns during Q1, Q2, and Q3 of the internship year were included in the current interim analysis. Baseline characteristics were recalculated directly from the Q1 records of these 42 interns and are reported in Table 3. There were 27 male participants (64.3%) and 15 female participants (35.7%). Most participants were Muslim (27/42, 64.3%), and 37 participants (88.1%) were born in Israel.

#### Internal Consistency of the Implemented Scores

Internal consistency varied by scale and quarter (Table 1). SWLS alpha was approximately 0.80 at each quarter. Organizational culture showed lower alpha values (0.573–0.678), as did work satisfaction at Q1–Q2 (0.583 and 0.576). Emotional- and physical-fatigue alpha values declined to 0.601 and 0.621 at Q3. These results do not support a blanket claim that all adapted measures had acceptable reliability or established validity.

### 3.2. Descriptive and Repeated-Measures Results

#### 3.2.1. Effort and Time Investment Domain

Self-reported weekly work hours were 58.98 ± 12.25 in Q1, 61.48 ± 11.51 in Q2, and 62.98 ± 11.82 in Q3 (*n* = 42). The omnibus time effect was not statistically significant (F[2,82] = 2.726, *p* = 0.071, partial η^2^ = 0.062). The linear contrast was nominally significant (F[1,41] = 4.954, *p* = 0.032, partial η^2^ = 0.108), with a Q3–Q1 increase of 4.00 h/week (95% CI 0.37–7.63). These tests address different hypotheses: the former tests any difference among three means, while the latter tests the ordered linear component. The nominal contrast does not establish a significant omnibus time effect.

Across Q1-3, participants rated their work effort at a 6.4 ± 0.8/7 (mean ± standard deviation across available questionnaires). On the standardized TC and WI scales, average time investment was rated 5.3 ± 1.0/7 and average work effort investment was 6.4 ± 0.7/7. Average sleep hours were reported to be 5.4 ± 1.0 h.

#### 3.2.2. Satisfaction and Motivation Domain

Across available questionnaires, work enjoyment was 4.9 ± 1.0/7, work drive was 5.5 ± 0.9/7, leisure satisfaction was 4.4 ± 1.5/7, general life satisfaction was 4.6 ± 1.2/7, and work satisfaction was 4.9 ± 1.0/7. For the primary SWLS analysis, 41 interns had valid scores at all three quarters. Their Q1, Q2, and Q3 means were 4.36 ± 1.24, 4.56 ± 1.18, and 4.72 ± 1.04, respectively. Mauchly’s test did not indicate a violation of sphericity (W = 0.995, *p* = 0.902). The omnibus effect did not reach statistical significance (F[2,80] = 3.005, *p* = 0.055, partial η^2^ = 0.070). The linear contrast was nominally significant (F[1,40] = 5.730, *p* = 0.021, partial η^2^ = 0.125); the Q3–Q1 mean difference was 0.36 points (95% CI 0.06–0.67). This positive contrast is a secondary description of the trajectory, not a positive primary omnibus result. Full repeated-measures results are provided in Table 2 and Table 4.

#### 3.2.3. Stress, Fatigue and Emotional Factors Domain

Mean perceived stress was 3.3 ± 0.8/7. Emotional and physical fatigue symptom indices were 2.2 ± 2.2/10 and 3.5 ± 2.4/10, respectively. Work-related positive affect was 4.7 ± 1.1/7 and negative affect was 3.3 ± 1.2/7. The significance/aptitude composite was 5.8 ± 0.9/7. Self-reported sleep duration was 5.4 ± 1.0 h. These pooled questionnaire-level summaries are descriptive; the longitudinal analyses use the outcome-specific complete samples shown in Table 2.

#### 3.2.4. Organizational and Financial Factors Domain

In 58.5% (72/123) of questionnaires, interns reported requesting extra overnight calls. (on top of the four calls required per month) due to financial issues. When presented with the hypothetical scenarios of winning the lottery or inheriting a large sum of money, that would make it possible for them to stop working, only 10/122 (8.2%) said they would actually stop working. In that event, the average work hours after acquiring that large sum of money would be 36.6 ± 15.2 (as compared to the mean self-reported work hours of 61.1 ± 11.9).

Participants rated the organizational work culture at 4.5 ± 1.2/7 on a scale of high-demand/work-intensive organizational culture.

#### 3.2.5. Complete Repeated-Measures Results

Table 2 reports the primary SWLS endpoint and all seven prespecified secondary repeated-measures outcomes. No omnibus test reached *p* < 0.05. Mauchly’s tests were nonsignificant for every outcome (all *p* ≥ 0.186), so sphericity-assumed degrees of freedom apply; Greenhouse–Geisser correction was not required. Table 4 separately reports all eight linear contrasts, including the nominally significant positive SWLS and work-hours contrasts. Confidence intervals are unadjusted and refer to the paired Q3–Q1 difference.

### 3.3. Analytic Statistics

#### 3.3.1. Associations of Age, Sex and Relationship Status

All analyses in this subsection used participant-level mean outcomes and baseline demographic characteristics. Older age was not significantly associated with time investment (Spearman’s rho = 0.175, *p* = 0.268; *n* = 42), work effort investment (rho = 0.243, *p* = 0.122; *n* = 42), or a high-demand organizational culture (rho = 0.216, *p* = 0.175; *n* = 41). Older age was negatively correlated with sleep hours (rho = −0.350, *p* = 0.023; *n* = 42). The association with the mean number of dependents did not reach nominal significance (rho = 0.284, *p* = 0.069; *n* = 42).

Sex comparisons included 27 males and 15 females, classified using their verified Q1 responses. Mean positive affect was 4.88 ± 0.87/7 versus 4.51 ± 1.02/7 (difference 0.37, 95% CI −0.27 to 1.01; *p* = 0.248). Perceived stress was 3.19 ± 0.69/7 versus 3.61 ± 0.48/7 (difference −0.42, 95% CI −0.79 to −0.05; *p* = 0.026). Physical fatigue was 2.97 ± 1.79/10 versus 4.44 ± 1.74/10 (difference −1.47, 95% CI −2.63 to −0.32; *p* = 0.014). Differences are male minus female; means and SDs summarize interns’ mean scores, and *p*-values are from Welch’s tests. Mann–Whitney sensitivity *p*-values for positive affect, stress, and physical fatigue were 0.189, 0.016, and 0.010, respectively. Requiring all three valid quarterly scores yielded *p* = 0.230, *p* = 0.010, and *p* = 0.029, respectively (27 males and 14 females for each comparison). After correction of the item-scoring error, the stress and physical-fatigue differences were nominally significant under both tests, whereas the positive-affect difference was not. No other tested sex comparison reached nominal significance using Welch’s test.

Relationship-status comparisons used verified Q1 status: 27 participants were single and 13 had a partner; two had missing baseline status and were excluded. Time investment was 5.39 ± 0.79/7 versus 4.99 ± 0.96/7 (difference 0.40, 95% CI −0.24 to 1.03; *p* = 0.210). Positive affect was 4.89 ± 0.93/7 versus 4.28 ± 0.78/7 (difference 0.61, 95% CI 0.04 to 1.18; *p* = 0.038). Work satisfaction was 5.07 ± 0.77/7 versus 4.69 ± 0.95/7 (difference 0.38, 95% CI −0.25 to 1.01; *p* = 0.221). Differences are single minus partnered. Partnered participants reported a higher-demand organizational culture (5.09 ± 0.71/7, *n* = 13, versus 4.19 ± 1.00/7, *n* = 26; partnered–single difference 0.90, 95% CI 0.33–1.46; *p* = 0.003). One single participant had only one valid culture score and was excluded from that comparison. Means and SDs summarize participant-level means. Mann–Whitney sensitivity *p*-values for time investment, positive affect, work satisfaction, and culture were 0.152, 0.048, 0.208, and 0.002, respectively. The positive-affect difference remained nominally significant when all three valid scores were required (*p* = 0.042; 26 single and 13 partnered participants). No other tested relationship-status comparison reached nominal significance using Welch’s test.

#### 3.3.2. Associations Within and Between Questionnaire Domains

Spearman correlations between participant-level mean scores described several nominal associations. All correlations in this subsection used 42 independent participants, except for organizational culture versus life satisfaction (*n* = 41). Time investment was positively correlated with effort investment (rho = 0.466, *p* = 0.002) and work enjoyment (rho = 0.586, *p* < 0.001). Effort investment was positively correlated with work drive (rho = 0.571, *p* < 0.001), life satisfaction (rho = 0.435, *p* = 0.004), positive affect (rho = 0.503, *p* < 0.001), and work satisfaction (rho = 0.324, *p* = 0.036). The significance/aptitude composite was positively correlated with time investment (rho = 0.595, *p* < 0.001) and effort investment (rho = 0.585, *p* < 0.001). These are descriptive between-participant associations without an inferred direction of influence.

Work enjoyment was positively correlated with work drive (rho = 0.570, *p* < 0.001), life satisfaction (rho = 0.385, *p* = 0.012), positive affect (rho = 0.615, *p* < 0.001), and work satisfaction (rho = 0.436, *p* = 0.004). Work drive was positively correlated with work satisfaction (rho = 0.312, *p* = 0.044) and positive affect (rho = 0.333, *p* = 0.031). The significance/aptitude composite was positively correlated with work enjoyment (rho = 0.461, *p* = 0.002) and positive affect (rho = 0.727, *p* < 0.001). Pearson sensitivity analyses did not reach nominal significance for enjoyment versus life satisfaction (r = 0.274, *p* = 0.079), work drive versus work satisfaction (r = 0.281, *p* = 0.072), or work drive versus positive affect (r = 0.269, *p* = 0.084). These weaker associations were method-dependent.

Positive affect was positively correlated with life satisfaction (rho = 0.578, *p* < 0.001) and work satisfaction (rho = 0.586, *p* < 0.001), and inversely correlated with stress (rho = −0.768, *p* < 0.001), emotional fatigue (rho = −0.561, *p* < 0.001), and negative affect (rho = −0.477, *p* = 0.001). Stress was inversely correlated with life satisfaction (rho = −0.402, *p* = 0.008) and work satisfaction (rho = −0.413, *p* = 0.007), and positively correlated with emotional fatigue (rho = 0.579, *p* < 0.001).

Within the organizational context, the association between high-demand/work-intensive organizational culture perception and life satisfaction did not reach nominal statistical significance in the participant-level analysis (rho = 0.297, *p* = 0.060; *n* = 41).

## 4. Discussion

This interim prospective repeated-measures study examined self-reported work investment, well-being, affective experience, fatigue, and organizational perceptions across the first three quarterly assessments of internship. The primary SWLS omnibus test was not statistically significant (*p* = 0.055), although an unadjusted positive linear contrast was observed (*p* = 0.021). The central finding is deliberately modest: within this small single-center sample, several measures that might have been expected to deteriorate during internship did not show statistically significant deterioration between Q1 and Q3. This finding should not be interpreted as proof that interns maintain stable satisfaction, motivation, or organizational perceptions throughout internship. Rather, it indicates that this interim dataset did not detect a clear decline across the measured outcomes during the early-to-middle part of the internship year.

### 4.1. Interpretation of Main Statistical Findings

The repeated-measures findings are best understood as preliminary evidence of no detected deterioration rather than evidence of durable resilience. Mean self-reported weekly work hours increased numerically across the three assessments, and the specific linear contrast was nominally significant, with a Q3–Q1 increase of 4.00 h/week (95% CI 0.37–7.63). However, the overall time effect did not reach statistical significance. A one-degree-of-freedom contrast tests a narrower hypothesis than the two-degree-of-freedom omnibus test, so these results are not contradictory. The contrast remains an unadjusted secondary finding, and the sample was not powered to exclude meaningful changes. Therefore, absence of statistically significant deterioration should not be equated with absence of burden.

In participant-level comparisons, male participants reported lower physical fatigue and stress than female participants after correction of the item-scoring error. The positive-affect difference was not statistically significant. The direction of these differences is consistent with the prior literature describing gender-related differences in stress and burnout among junior physicians and medical professionals [12,13,14,15]. Nevertheless, the present comparisons were exploratory, based on small subgroup sizes, and not corrected for multiplicity. They should therefore be viewed as preliminary between-participant signals that warrant further study rather than definitive evidence of sex-specific effects.

Baseline relationship status was associated with selected affective and organizational measures. Single participants reported higher average work-related positive feelings, whereas participants with partners perceived the organizational culture as more demanding. Differences in average time investment and work satisfaction were not statistically significant after participant-level aggregation. These results may reflect differences in external responsibilities, work–life boundaries, or perceived role strain. However, they are correlational and may also be influenced by unmeasured confounding factors such as financial pressures, family obligations, department placement, and individual preferences for work intensity.

The correlations between participant-level mean scores described associations among investment, enjoyment, work drive, affect, satisfaction, and the self-reported significance/aptitude composite. This pattern is compatible with the heavy work investment framework, in which high investment may reflect both adaptive engagement and potentially maladaptive work drive [5,6,7,17]. However, the correlation matrix was exploratory and included many comparisons. These associations describe differences between interns and do not demonstrate that changes in one domain track changes in another within the same intern. As a result, the observed associations should be used to refine hypotheses and analytic priorities for a future full-cohort analysis rather than to support strong mechanistic conclusions.

Work-related positive emotions were inversely associated with stress, emotional fatigue, and negative emotions and positively associated with life and work satisfaction. These relationships were descriptive, and the present design cannot establish whether positive affect protects against stress, whether lower stress permits more positive affect, or whether both are shaped by shared individual and organizational factors.

The association between perceived high-demand organizational culture and life satisfaction did not reach nominal significance after participant-level aggregation. Accordingly, this analysis does not provide clear evidence for the previously suggested positive association. Person-environment fit, selection, perceived institutional support, and unmeasured confounding remain possible topics for future investigation, but cannot be inferred from this result. It should not be read as evidence that high organizational demands improve well-being.

Taken together, in this interim cohort, work investment, satisfaction, affect, fatigue, and organizational perception appear interrelated, but the study cannot determine causality, long-term stability, or generalizability beyond this setting.

### 4.2. Comparison with the Existing Literature

The present findings can be considered alongside previous research on correlates of work investment and well-being among professionals. Although previous work describes escalating work hours and responsibilities during medical training (1,4), the revised participant-level analysis did not detect significant age associations with time or effort investment. The direction of the sex differences in fatigue and stress is compatible with findings from both Australian and American cohorts, where women report higher stress and burnout despite similar levels of work engagement (3).

Relationship status has been less extensively examined in relation to work investment and satisfaction. In this cohort, baseline single status was associated with greater average positive affect, whereas partnered status was associated with higher perceived organizational demands. There was no statistically significant difference in time investment or work satisfaction. Competing personal responsibilities and role tensions are possible hypotheses for future investigation (2); they were not tested as explanations in this study.

The exploratory correlations among investment, enjoyment, work drive, and positive affect are compatible with the distinction between the engaged and compulsive forms of heavy work investment described by Snir and Harpaz (2012) [5]. They do not establish distinct subtypes, demonstrate adaptive versus maladaptive consequences, or confirm this theoretical duality. No causal pathways were tested.

The relationship between organizational culture and life satisfaction remains uncertain in this dataset because the revised participant-level association was not statistically significant. Models of person-environment fit, which posit that alignment between individual preferences and organizational characteristics matters for well-being, may inform future research, but the present analysis does not establish that organizational intensity is rewarding.

### 4.3. Implications for Theory and Practice

The findings motivate the further study of relationships among individual characteristics, affect, work investment, and organizational perceptions. Whether interventions that enhance motivation or emotional well-being improve outcomes is a separate hypothesis requiring intervention studies. The present associations provide no estimate of intervention benefit or evidence that work drive causes adverse outcomes.

For educational leaders and hospital administrators, these exploratory results may help formulate questions about support needs during internship. Potential differences by sex and baseline relationship status warrant replication in larger cohorts with appropriate adjustment for confounding. This study does not establish that tailored support, organizational changes, or safeguards improve satisfaction, performance, burnout, or retention.

The inverse associations of positive affect with stress and fatigue may inform hypotheses about engagement and recognition. Whether promoting meaning, perceived contribution, or efficacy reduces stress and fatigue remains untested. Reverse causation and shared individual or organizational influences are alternative explanations.

### 4.4. Methodological Strengths and Limitations

The study has several strengths. It used repeated measurements within the same interns rather than relying on a single cross-sectional snapshot, and it incorporated items informed by published instruments across multiple domains relevant to internship experience, with the composition and internal consistency of the implemented scores now reported explicitly. The repeated-measures structure allowed the analysis to examine within-participant change across quarterly assessments, while the domain-based questionnaire enabled exploration of relationships between work investment, satisfaction, affect, fatigue, and organizational perceptions.

Several limitations are important. First, this was an interim single-center repeated-measures analysis with only 42 participants, and three completed quarterly measurements. The sample size limits statistical power, precision, and generalizability. Participant-level aggregation removes the independence violation in exploratory inference, but summarizes experience over Q1–Q3 and cannot resolve within-participant associations or time-dependent effects. Available-score means may also be affected by missing data. Second, the analysis included multiple secondary group comparisons and correlations without formal multiplicity correction. Consequently, nominally significant secondary associations may include false-positive findings and should be considered exploratory. Third, reliance on self-report introduces possible recall, response, and social desirability biases, particularly for sensitive constructs such as stress, fatigue, work drive, financial pressure, and satisfaction. Internal consistency was limited for organizational culture and for work satisfaction at Q1–Q2, and the fatigue indices had lower alpha values at Q3. The exact adapted scores, including the fatigue and significance/aptitude indices, have not undergone independent construct or criterion validation in this intern population. Reliability does not establish validity, and longitudinal measurement invariance was not tested; these limitations constrain comparisons and interpretation.

Fourth, the study did not fully account for potential confounders such as department rotation, specialty preference, socioeconomic status, baseline mental health, institutional support exposure, or prior clinical experience. Finally, the absence of statistically significant deterioration in several outcomes does not prove stability, resilience, or absence of harm; it may reflect limited power, measurement timing, adaptation, selection, or unmeasured contextual factors.

The present findings should therefore be used primarily to guide a more focused, prespecified analysis in the complete cohort.

### 4.5. Suggestions for Future Research

Building on these findings, future research should prioritize longitudinal designs to clarify causal pathways and temporal dynamics. Studies that incorporate objective measures of work investment (e.g., hours worked, productivity metrics) alongside subjective assessments may yield a more comprehensive understanding of work-related behaviors. Additionally, expanding the scope to include diverse occupational groups and organizational contexts could enhance generalizability.

Qualitative investigations may illuminate the lived experiences and coping strategies of professionals navigating high-demand environments, particularly in relation to gender and relationship status. Interventional studies assessing the efficacy of programs targeting motivation, emotional well-being, and work–life balance would provide valuable evidence for organizational policy and practice.

Finally, research exploring the moderating and mediating roles of organizational culture, social support, and individual resilience could refine theoretical models and inform targeted interventions. The integration of person-environment fit perspectives may be especially fruitful in understanding the conditions under which high work investment is adaptive versus maladaptive.

## 5. Conclusions

In this interim prospective repeated-measures study of 42 medical interns at a single tertiary medical center, the omnibus repeated-measures test did not detect a statistically significant change in the prespecified primary endpoint of general life satisfaction (*p* = 0.055). A positive linear contrast was nominally significant (*p* = 0.021) and is interpreted as secondary to the omnibus test. Several secondary self-reported measures of work enjoyment, stress, fatigue, work satisfaction, weekly work hours, and organizational perceptions likewise did not show statistically significant deterioration across Q1–Q3. These results should be interpreted as absence of detected deterioration in a small interim sample, not as definitive evidence that satisfaction, motivation, or organizational perceptions are sustained throughout internship.

Exploratory analyses of participant-level mean scores suggested lower stress and physical fatigue among males, associations of baseline relationship status with positive affect and organizational perceptions, and several between-participant associations among work investment, affect, satisfaction, stress, and fatigue. The organizational-culture–life-satisfaction association was not statistically significant. Because these analyses involved multiple comparisons and correlations without formal multiplicity correction, they should be considered hypothesis-generating.

For educational leaders and hospital administrators, the study underscores the need to monitor interns’ well-being during the internship year.

## Figures and Tables

**Table 1:** Item composition, scoring, and quarterly internal consistency.

Score	k	Item Identifiers	Scoring	Q1 α (*n*)	Q2 α (*n*)	Q3 α (*n*)
Time investment [16]	5	Q3.1–Q3.5	Mean (1–7)	0.790 (42)	0.826 (42)	0.744 (41)
Effort investment [16]	5	Q3.6–Q3.10	Mean (1–7)	0.645 (42)	0.777 (42)	0.883 (42)
Work enjoyment [17]	7	Q7.1–Q7.7	Mean; reverse Q7.6	0.721 (41)	0.796 (42)	0.779 (42)
Work drive [17]	7	Q7.8–Q7.14	Mean (1–7)	0.699 (42)	0.730 (42)	0.836 (42)
Organizational culture [18]	3	Q8.1–Q8.3	Mean (1–7)	0.573 (41)	0.678 (41)	0.677 (41)
Life satisfaction [19]	5	Q9.1–Q9.5	Mean (1–7)	0.799 (42)	0.797 (41)	0.797 (42)
Positive affect [20]	6	Q10.1, 10.4, 10.5, 10.7, 10.9, 10.10	Mean (1–7)	0.792 (40)	0.821 (40)	0.838 (42)
Negative affect [20]	6	Q10.2, 10.3, 10.6, 10.8, 10.11, 10.12	Mean (1–7)	0.790 (40)	0.846 (40)	0.797 (41)
Work satisfaction [21]	4	Q11.1–Q11.4	Mean (1–7)	0.583 (41)	0.576 (41)	0.723 (42)
Perceived stress [22]	14	Q12.1–Q12.14	Mean; 7 items reversed *	0.830 (37)	0.802 (37)	0.769 (41)
Emotional fatigue [23]	10	Q13.1–Q13.10	10 × mean of binary items (0–10)	0.780 (39)	0.813 (40)	0.601 (39)
Physical fatigue [23]	10	Q13.11–Q13.20	10 × mean of binary items (0–10)	0.826 (40)	0.785 (41)	0.621 (41)
Significance and aptitude	6	Q16.1–Q16.6	Mean (1–7)	0.842 (40)	0.920 (40)	0.937 (42)

k, number of items. * Stress items Q12.4, 12.5, 12.6, 12.7, 12.9, 12.10, and 12.13 were reversed as 8-response; the same reversal was used for Q7.6. All Likert composites are means on a 1–7 scale. Alpha uses complete item sets separately within each quarter; *n* is the number of independent interns contributing to that estimate. Fatigue alpha is equivalent to KR-20. Scores may use available valid items, so reliability *n* can be smaller than the longitudinal analysis *n*. References indicate conceptual/item sources, not validation of the exact study implementation. Significance/aptitude and fatigue are treated as study-specific composites.

**Table 2:** Quarterly means and omnibus repeated-measures ANOVA.

Outcome	*n*	Q1 Mean ± SD	Q2 Mean ± SD	Q3 Mean ± SD	F (df1, df2)	*p*	Partial η^2^
Life satisfaction	41	4.36 ± 1.24	4.56 ± 1.18	4.72 ± 1.04	3.005 (2, 80)	0.0551	0.070
Work satisfaction	42	5.05 ± 0.92	4.91 ± 0.94	4.87 ± 1.08	0.844 (2, 82)	0.4338	0.020
Work enjoyment	42	4.90 ± 0.95	4.85 ± 1.02	4.88 ± 0.95	0.103 (2, 82)	0.9026	0.002
Perceived stress	41	3.29 ± 0.77	3.42 ± 0.73	3.34 ± 0.76	0.802 (2, 80)	0.4521	0.020
Emotional fatigue	41	2.01 ± 2.24	2.15 ± 2.44	2.35 ± 2.04	0.539 (2, 80)	0.5853	0.013
Physical fatigue	41	3.13 ± 2.64	3.74 ± 2.53	3.35 ± 2.04	1.066 (2, 80)	0.3493	0.026
Weekly work hours	42	58.98 ± 12.25	61.48 ± 11.51	62.98 ± 11.82	2.726 (2, 82)	0.0714	0.062
Organizational culture	41	4.28 ± 1.16	4.68 ± 1.19	4.47 ± 1.30	2.156 (2, 80)	0.1225	0.051

SWLS is the primary endpoint; the remaining rows are prespecified secondary outcomes. Each row uses the same complete-case participants at Q1, Q2, and Q3. Scores are 1–7 except fatigue indices (0–10) and work hours (hours/week). Partial η^2^ describes the time effect. Degrees of freedom are uncorrected because no outcome violated sphericity at the 0.05 level. All *p*-values are two-sided where applicable and unadjusted for multiplicity.

**Table 3:** Baseline demographic characteristics of the final analytic cohort (*n* = 42).

Variable	*n* (%)
Age (years, mean (SD))	28.3 (3.1)
Sex	
Male	27 (64.3%)
Female	15 (35.7%)
Religion	
Muslim	27 (64.3%)
Jewish	8 (19.0%)
Other	7 (16.7%)
Country of Birth	
Israel	37 (88.1%)
Former Soviet Union	5 (11.9%)
Birth Order	
Firstborn	1 (2.4%)
Second born	15 (35.7%)
Third-born	18 (42.9%)
Fourth-born	8 (19.0%)
Relationship Status	
Has a partner	13 (31.0%)
Single	27 (64.3%)
Missing	2 (4.8%)
Dependents	
0	2 (4.8%)
1	15 (35.7%)
2	9 (21.4%)
3 and above	16 (38.1%)

SD, standard deviation. Percentages use all 42 participants as the denominator; missing relationship status is shown explicitly.

**Table 4:** Linear contrasts and paired Q3–Q1 differences.

Outcome	*n*	Linear F (df1, df2)	*p*	Partial η^2^	Q3–Q1 Difference (95% CI)
Life satisfaction	41	5.730 (1, 40)	0.0215	0.125	0.36 (0.06 to 0.67)
Work satisfaction	42	1.245 (1, 41)	0.2710	0.029	−0.18 (−0.51 to 0.15)
Work enjoyment	42	0.035 (1, 41)	0.8529	0.001	−0.02 (−0.26 to 0.22)
Perceived stress	41	0.201 (1, 40)	0.6561	0.005	0.05 (−0.16 to 0.26)
Emotional fatigue	41	1.437 (1, 40)	0.2377	0.035	0.34 (−0.23 to 0.91)
Physical fatigue	41	0.253 (1, 40)	0.6178	0.006	0.22 (−0.67 to 1.12)
Weekly work hours	42	4.954 (1, 41)	0.0316	0.108	4.00 (0.37 to 7.63)
Organizational culture	41	1.004 (1, 40)	0.3224	0.024	0.19 (−0.19 to 0.58)

Linear contrast coefficients are −1, 0, and +1. With three equally spaced assessments, this contrast is proportional to Q3–Q1; F equals the squared paired t statistic. Partial η^2^ = F/(F + n − 1). Confidence intervals are paired t-based intervals in the score’s original units. All contrasts are nominal, unadjusted secondary analyses; none replaces the primary omnibus SWLS test.

## Data Availability

Research data will be available upon request from the corresponding author, under the restrictions and regulations of the institutional IRB.
